# The concordance between upper and lower respiratory microbiota in children with *Mycoplasma pneumoniae* pneumonia

**DOI:** 10.1038/s41426-018-0097-y

**Published:** 2018-05-23

**Authors:** Wenkui Dai, Heping Wang, Qian Zhou, Xin Feng, Zhiwei Lu, Dongfang Li, Zhenyu Yang, Yanhong Liu, Yinhu Li, Gan Xie, Kunling Shen, Yonghong Yang, Yuejie Zheng, Shuaicheng Li

**Affiliations:** 1 Department of Computer Science, City University of Hong Kong, 999077 Hong Kong, China; 2 Department of Respiratory Diseases, Shenzhen Children’s Hospital, No. 7019, Yitian Road, 518026 Futian District, Shenzhen China; 3 Department of Microbial Research, WeHealthGene Institute, 3C19, No. 19 Building, 518000 Dayun Software Town, Shenzhen China; 4 Institute of Statistics, NanKai University, No. 94 Weijin Road, 300071 Tianjin, China; 5 Department of Respiratory Diseases, Beijing Children’s Hospital, 100045 Beijing, China

## Abstract

In recent years, the morbidity of *Mycoplasma pneumoniae* pneumonia (MPP) has dramatically increased in China. An increasing number of studies indicate that an imbalance in the respiratory microbiota is associated with respiratory infection. We selected 28 hospitalized patients infected with *M. pneumoniae* and 32 healthy children. Nasopharyngeal (NP) and oropharyngeal (OP) swabs were collected from healthy children, whereas NP, OP and bronchoalveolar lavage (BAL) specimens were collected from patients. Microbiota analysis was performed on all microbial samples using 16 S ribosomal RNA (16 S rRNA) sequencing. The NP microbial samples in healthy children were divided into two groups, which were dominated by either *Staphylococcus* or mixed microbial components. The respiratory microbiota in pneumonia patients harbored a lower microbial diversity compared to healthy children, and both the NP and OP microbiota of patients differed significantly from that of healthy children. Hospitalized MPP children with a higher abundance of *Mycoplasma* in the BAL fluid (BALF) microbiota tended to suffer longer hospitalization lengths and higher peak fevers and serum C-reactive protein levels. Concordance analysis explained the succession of imbalanced NP microbiota to the OP and lung in diseased children. However, the association of the abundance of *Mycoplasma* in BALF microbiota with that in NP or OP microbiota varied among individuals, which suggested the sensitivity of BALF in MPP diagnostics, mirroring MPP severity.

## Introduction

The respiratory tract is home to a variety of microbial commensals that cultivate the immune system and confer resistance to colonization of pathogens^1, 2^. Increasing reports have demonstrated the association of altered respiratory microbiota with disease severity in acute respiratory infections (ARIs), such as bronchiolitis and pneumonia^3–5^. *Mycoplasma pneumoniae* pneumonia (MPP) is resulting in an increasingly high morbidity in Chinese children^6^. Previous studies have also demonstrated the alterations of the nasopharynx (NP) and oropharynx (OP) microbiota in MPP, as well as microbiota transmission between the NP and OP^5, 7, 8^.

However, imbalanced lung microbiota in MPP remains unexplored. Meyer Sauteur PM *et al.* showed that the clearance of *M. pneumoniae* in the lung differed significantly from that in the NP^9^, which suggested different alterations of lung microbiota in MPP compared to the NP/OP. In addition, the use of bronchoalveolar lavage fluid (BALF) is a more sensitive measure for the establishment of microbial diagnosis^10^ and assessment of disease severity compared with NP and OP swabs^11, 12^. Given that BALF collection is restricted to cases of severe disease, it is imperative to explore the possibility of predicting lung microbiota alterations via NP or OP microbiota analysis.

In this study, we enrolled 28 MPP and 32 healthy children to analyze respiratory microbiota via 16 S rRNA analysis. We intended to answer the following two questions: 1) how the imbalanced NP/OP microbiota is associated with lung microbiota in MPP and 2) how altered respiratory microbiota is associated with MPP severity.

## Results

### Sample characteristics

Both healthy and diseased children were not exposed to antibiotics for at least one month prior to sampling to ensure no confounding effect of antibiotics on the respiratory microbiota. None of the recruited healthy children suffered any chronic respiratory diseases or acute respiratory infection during the one month prior to sampling and for one week after sampling. Selected patients had chest radiographic abnormalities consistent with pneumonia (Table 1). Notably, the average hospitalization length of MPP children with a history of pneumonia was longer than that of other patients (Table S1).

**Table 1:** Sample information

	Pneumonia Patients (*n* = 28)	Healthy Children(*n* = 32)
Characteristics		
Gender		
Female	12	16
Male	16	16
Age (years)	4.5(0.7–10.5)	4.0(0.3–9.9)
Delivery mode		
Cesarean section	12	9
Vaginally born	16	23
Feed pattern		
Breast feed	17	11
Breast feedmilk feed	5	15
Milk feed	6	6
Family history of allergy	0	0
History of pneumonia	4	0
Asthma	0	0

Clinical records		
Lung consolidation, atelectasis, infiltration	28	NA
Hospitalization time (days)	9.5(4–37)	–
Fever	20	–
Cough	25	–
Wheezing	2	–
CRP (<0.499mg/l)	8	NA
PCT (<0.5ng/ml)	28	NA
Eosinophil (0.5–5%)	15	NA

”-“ represents not detected, *NA* represents not available, *CRP* C-response protein, *PCT* procalcitonin

### Data output and confounder assessment

A total of 8,177,030 high-quality tags were produced, averaging 41,846 (27,666–56,342), 30,848 (15,558–53,235), 67,467 (25,505–77,144), 73,856 (45,551–77,154) and 67,634 (19,862–77,454) for the NP-healthy (NP-H), OP-healthy (OP-H), NP-pneumonia (NP-P), OP-pneumonia (OP-P), and BALF groups, respectively. The average OTU numbers in the NP-H, OP-H, NP-P, OP-P, and BALF groups were 460, 343, 242, 134, and 763, respectively. Association analysis indicated that the onset of pneumonia most significantly explained the variations in microbial samples (*p*-value < 0.001) (Table S2). The concentration of extracted DNA in the unused sampling swabs and DNA extraction kits was lower than 0.01 ng/μl, whereas it was higher than 80 ng/μl in the sampling swabs and BALF. In addition, 16 S rRNA gene amplification on the extracted DNA indicated less than 0.01 nmol/l bacterial DNA in the enveloped sampling or extraction materials.

### The alterations of NP and OP microbiota in MPP patients

**Fig. 1:**
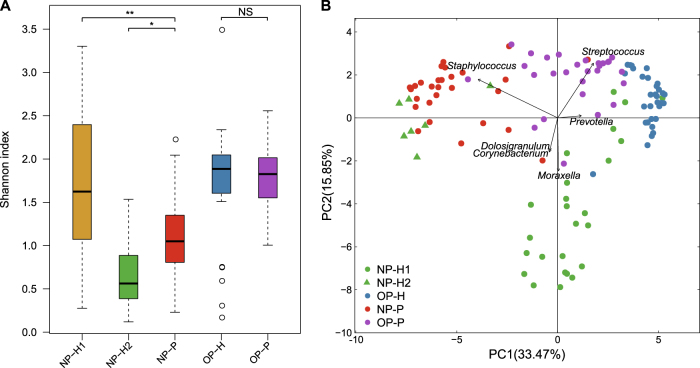
Nasopharyngeal (NP)/oropharyngeal (OP) microbiota structure in *Mycoplasma Pneumoniae* pneumonia (MPP) patients and healthy children. **a** Shannon index of NP and OP microbiota in patients and healthy infants; healthy NP samples were further divided into NP-H1 and NP-H2. Asterisk (*) and (**) represent *p*-values of ≤0.05 and ≤0.01. NS represents not significant. **b** Principal component analysis (PCA) of NP and OP samples. Points colored green, blue, red and purple represent samples from NP-H1, NP-H2, OP-H, NP-P, and OP-P, respectively

There was no difference between bacterial diversity in the OP microbiota of MPP patients and that of healthy children (Fig. 1a). NP microbial samples in healthy children were divided into two clusters based on principal component analysis (PCA) (Fig. 1b), whereas OP microbial samples in healthy and diseased children were clearly separated (Fig. 1b). In parallel, the bacterial richness and evenness in the NP microbiota of the two clusters differed significantly in healthy children (Fig. 1a).

**Fig. 2:**
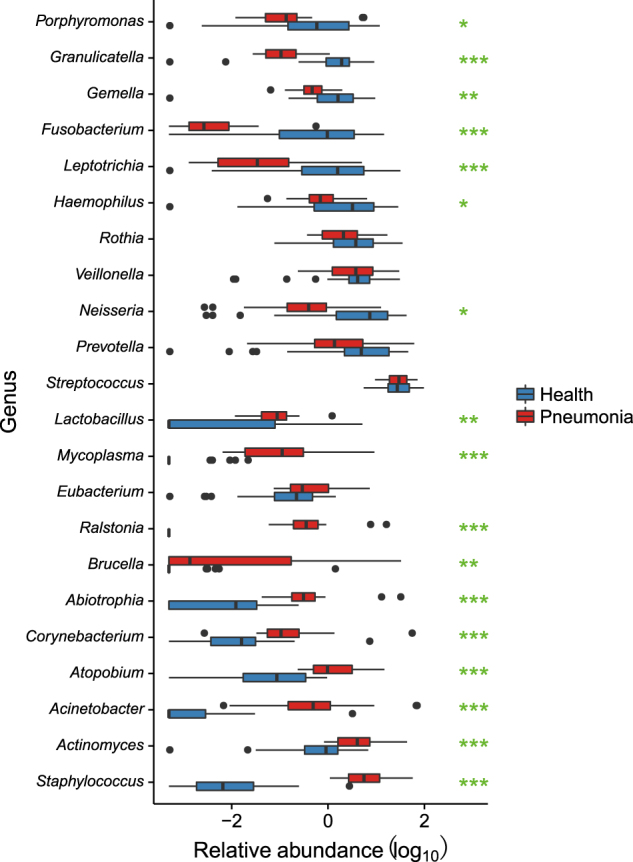
Comparison of oropharyngeal (OP) dominant genera between patients and healthy children. The vertical axis represents the genus name, and the horizontal axis shows the log10 value of relative abundance. Asterisk (*), (**), and (***) represent *q*-values of ≤0.05, ≤0.01, and ≤0.001, respectively

Compared to the other twenty-five NP-H samples (NP-H1), we identified a lower bacterial diversity in seven NP-H samples (NP-H2) (Fig. 1a), which were clustered in the NP-P group (Fig. 1b, *p*-value < 0.01). Further analysis indicated that *Staphylococcus* dominated in the NP-H2 (81.87%) and NP-P (62.28%) microbiota, whereas NP-H1 microbiota mainly comprised *Moraxella* (21.66%), *Streptococcus* (11.97%), *Corynebacterium* (11.44%) and *Dolosigranulum* (10.22%) (Fig. S1, Table S3). Conversely, the OP microbiota structure was similar in all healthy children (Table S3), comprising highly abundant *Streptococcus* (35.94%, vs. 31.32% in the OP-P microbiota, *q*-value = 0.93) and *Prevotella* (12.43%, vs. 6.82% in the OP-P microbiota, *q*-value = 0.09) (Fig. 2). In addition, *Staphylococcus*, *Corynebacterium* and *Mycoplasma* were significant components in the OP-P microbiota (Fig. 2).

### Significant association of lung microbiota with disease severity in MPP patients

**Fig. 3:**
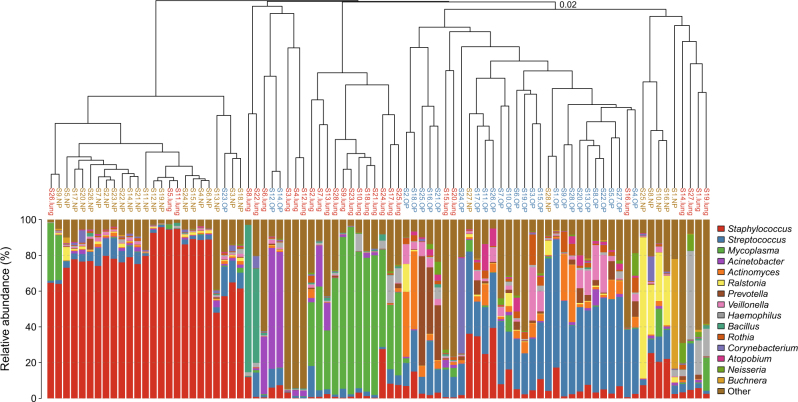
Dendrogram and dominant genus bar plot based on the hierarchical clustering of pneumonia samples. Clustering was based on the Bray-Curtis dissimilarity distance. The clade names colored yellow, blue and red represent samples originating from the NP, OP and lung, respectively

The hierarchical cluster analysis (Fig. 3) showed that one cluster was composed of only 12 BALF samples (BALF-1) (Fig. 3), with *Mycoplasma* being dominant (61.40%, 32.91–93.69%) in the lung microbiota (Table S4). Significantly decreased levels of *Mycoplasma* (4.08%, 0.12–32.24%) were verified in the lung microbiota in the remaining 16 BALF samples, which were identified in the NP or OP clusters (BALF-2) and featured *Haemophilus*, *Staphylococcus* or novel microbial genera as the dominant isolates in the lung microbiota (Fig. 3). The bacterial diversity in BALF-1 is higher than that in BALF-2, but the difference is not significant (Figure S2).

The peak fever was higher in BALF-1 patients than in BALF-2 patients (40.2 °C vs. 39.4 °C, *p*-value < 0.05) (Table S1). The length of hospitalization and length of persistent fever were also longer in BALF-1 patients (average 12.8 days and 14.4 days) compared with the BALF-2 (average 9.8 days and 7.1 days) group (*p*-value = 0.267 and <0.05). In addition, we identified a higher abnormal CRP ratio in the BALF-1 group (10/12 in BALF-1, 6/16 in BALF-2) (Table S1). Subject S10, who previously had pneumonia, suffered the longest hospitalization period (37 days) and had an extremely high level of serum CRP (121.2 mg/l) during hospitalization, which may be caused by bilateral pulmonary effusion and necrotizing lung consolidation (Table S1). This can be attributed to severe *M. pneumoniae* infection, with 76.39% of the lung microbiota being *Mycoplasma* (Table S4), and exposure to various empirical antibiotics (Table S1).

### Similarity of microbial communities between respiratory niches in MPP patients

**Fig. 4:**
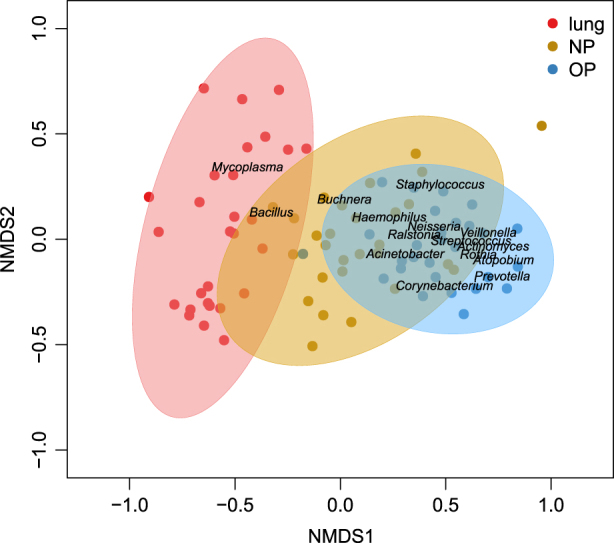
Two-dimensional non-metric multidimensional scaling (nMDS) plot of the microbial community composition in the nasopharyngeal (NP), oropharyngeal (OP) and bronchoalveolar lavage (BAL) samples. Dots colored yellow, blue and red represent samples originating from the NP, OP and lung respectively. Ellipses around the geometric mean are the standard deviations of niche samples

The similarity between upper respiratory tract (URT) and lower respiratory tract (LRT) microbiota was assessed to determine whether URT microbiota could mirror or predict microbiota imbalances in the lung. A non-metric multidimensional scaling (nMDS) analysis showed that the lung microbiota was more similar to that of the NP than the OP (Fig. 4). Additionally, NP microbial samples were clustered between BALF and OP samples (Fig. 4), which indicated the transmission of NP microbiota to both the lung and OP.

Of the top fifteen genera in URT and LRT microbiota, the abundances of *Staphylococcus*, *Corynebacterium* and *Haemophilus* were not different between the OP and lung microbiota (Table 2, Fig. S3). By comparison, we observed a decrease in *Streptococcus* and *Prevotella* in both the NP and lung microbiota (Table 2). *Mycoplasma* dominated the BALF microbiota (28.64%), which, however, was in low abundance in the URT (1.96% in NP and 0.60% in OP microbiota) (Table 2). *Ralstonia* and *Atopobium* represented ≤0.05% of the lung microbiota, whereas *Acinetobacter* and *Buchnera* were not significantly different among the NP, OP and lung (Table 2).

**Table 2:** Comparison of top 15 genera between the URT and LRT in MPP patients

Genus	*q*-value	Nemenyi post hoc test *p*-value		Relative abundance %		
	Kruskal test of 3 sites	NP-lung	OP-lung	lung	NP	OP
*Staphylococcus*	**<0.001**	**<0.001**	0.276	12.26	62.28	10.71
*Corynebacterium*	**0.005**	**0.008**	0.871	0.43	1.28	2.11
*Streptococcus*	**<0.001**	0.257	**<0.001**	6.13	9.74	31.32
*Mycoplasma*	**<0.001**	**<0.001**	**<0.001**	28.64	1.96	0.60
*Acinetobacter*	0.684	0.977	0.823	3.44	1.02	5.57
*Actinomyces*	**<0.001**	**0.026**	**<0.001**	0.44	0.73	8.41
*Ralstonia*	**<0.001**	**<0.001**	**<0.001**	0.03	7.13	1.16
*Prevotella*	**<0.001**	0.937	**<0.001**	0.43	0.42	6.82
*Veillonella*	**<0.001**	**<0.001**	**<0.001**	0.19	0.86	6.25
*Haemophilus*	**0.007**	**0.014**	0.969	4.92	0.56	1.17
*Bacillus*	**<0.001**	**<0.001**	**<0.001**	5.52	0.14	0.13
*Rothia*	**<0.001**	**0.018**	**<0.001**	0.39	0.54	3.27
*Atopobium*	**<0.001**	**<0.001**	**<0.001**	0.02	0.31	2.40
*Neisseria*	**0.009**	0.726	0.090	1.09	0.16	1.33
*Buchnera*	0.496	0.566	1.000	0.09	2.37	0.07

*p* < 0.05 or *q* < 0.05 are shown in bold

### Individual-specific NP and OP microbiota compared to the BALF

**Fig. 5:**
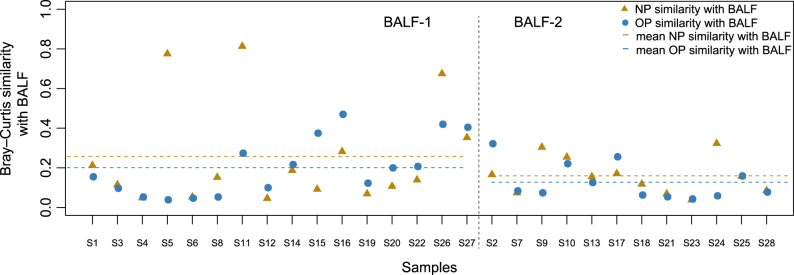
Intra-individual concordance between the nasopharyngeal (NP)/oropharyngeal (OP) microbiota and paired bronchoalveolar lavage fluid (BALF) microbiota. The 16 subjects on the left represent patients according to BALF-2, and the 12 subjects on the right represent patients according to BALF-1. Points colored blue are the similarity indices between the NP and BALF, and triangles colored yellow are the similarity indices between the OP and BALF

Bray–Curtis similarity measures were applied to assess the concordance between the NP, OP and BALF microbiota in hospitalized children. We observed variable intra-individual concordance between NP/OP and BALF, and the similarity between the NP and BALF microbiota was higher than that between the OP and BALF microbiota (Fig. 5). In the BALF-1 group, we identified a higher similarity between the NP and BALF microbiota and the OP and BALF microbiota compared with the BALF-2 group (Fig. 5). In parallel, the abundance of *Mycoplasma* was higher in the NP and OP microbiota of patients in the BALF-1 group compared to the BALF-2 group (Fig. S2, Table S4). For two individuals, S5 and S11 in the BALF-1 group, we identified *Staphylococcus* as being dominant (94.50% and 94.81%, respectively) in the lung microbiota, as well as a high similarity between the lung and NP microbiota (Fig. 5, Table S4). The present clinical tests identified *M. pneumoniae* in BALF samples even with 0.12% *Mycoplasma* (patient S4) in the lung microbiota (Table S4), which suggested the accuracy and sensitivity of the current MPP diagnostics based on BALF. However, it is difficult to establish an *M. pneumoniae* diagnosis by NP or OP swab, given that *M. pneumoniae* represented less than 0.05% of the URT microbiota in individuals with 1.35–54.52% *M. pneumoniae* in the lung microbiota (Table S4).

## Discussion

Emerging reports have demonstrated the pivotal role of respiratory microbiota in immune system education and resistance to colonization by pathogenic organisms^2, 13^. Several studies revealed various patterns of the NP microbiota in healthy children, with different susceptibilities to ARI^14–16^. Our study also identified the divergence of NP microbiota in healthy children, which was dominated by either *Staphylococcus* or mixed microbial components. A *Staphylococcus*-dominated NP microbiota pattern was reported to be associated with high severity of disease in ARIs, whereas a mixed NP microbiota pattern suggested a lower ARI risk^3, 17, 18^. These differences may explain the clustering of the seven microbial samples with a *Staphylococcus*-dominated NP microbiota in the NP-P group, and it may also guide the comparison of NP microbiota structures between the two healthy groups (NP-H1 and NP-H2) and the patient group (NP-P). With only a one-week follow-up after sampling, we identified no differences in the clinical characteristics between the NP-H1 and NP-H2 groups. In contrast to the two NP groups identified, the OP microbiota in healthy children showed no significant difference. The respiratory commensals decreased significantly in MPP, including a reduced amount of the lactic acid-producing bacterium *Lactobacillus* and the short-chain fatty acid producer *Porphyromonas*^19, 20^. *Prevotella*, which antagonizes lipid polysaccharides and inhibits mucosal inflammation, was also decreased^21^. This may provide clues about the microbial etiology in MPP at the level of the microbiota.

Food ingestion and esophageal reflux affected the oropharyngeal microbial composition, which signifies a higher bacterial richness in the OP than in NP and lung^11^. Prevaes *et al.*demonstrated that BALF microbial samples clustered between the NP and OP, showing a higher similarity between the lung and NP microbiota than between the lung and OP microbiota in children with cystic fibrosis^22^. A prospective study of 112 infants indicated that there was exchange between the NP and OP microbiota in children with high ARI risk in early life^23^. Our study identified the clustering of NP microbial samples between BALF and OP samples, which suggested the transmission of NP microbiota to both the OP and the lung. This may be due to nasal infection and robust pathogenic intrusion of *M. pneumoniae*^24^, which also induced the disequilibrium of OP microbiota in diseased children. Correspondingly, the NP-lung axis should be explored further to identify *M. pneumoniae* infections and the associated microbiota imbalance, as well as host responses.

Notably, MPP patients can be divided into two groups that feature different levels of *Mycoplasma* abundance in the lung microbiota. Consistent with previous studies that indicated that the abundance of either *Lactobacilli*,* Rothia* or *S. pneumoniae* was related to the pneumonia severity index (PSI)^4^, children with a higher load of *Mycoplasma* in the lung microbiota tended to suffer longer hospital stays, higher peak fevers and abnormal CRP ratios. Compared to patients with a low abundance of *Mycoplasma* in the lung microbiota, the average proportion of *Mycoplasma* in NP and OP microbiota was also greater in patients with a high abundance of *Mycoplasma* in the lung. Nonetheless, the *Mycoplasma* load in the NP and OP microbiota was not proportional to that in the lung microbiota at the individual level, suggesting the limitations of using URT microbiota to predict MPP severity. This could be explained by the differences in clearance of *M. pneumoniae* in the lung compared to the URT^9^. The complex etiology in pneumonia, such as co-infection with unknown pathogens, may explain the long hospitalization lengths and fever durations in patients with a low abundance of *M. pneumoniae* in the BALF.

Several limitations of this study should also be considered. The short-term follow-up for healthy children made it difficult to assess the susceptibility of different URT microbiota to ARI. The small sample size could impose restrictions on determining the association of respiratory microbiota with disease severity. Moreover, we could neither precisely identify the pathogens at the species level nor reveal functional dysbiosis in the respiratory microbiota in MPP via 16 S rRNA analysis.

In conclusion, we provide significant evidence for an imbalanced URT microbiota in MPP and varied URT microbiota patterns in healthy children. More importantly, we explored the transmission of URT and LRT microbiota, which suggested an association of respiratory microbiota imbalance with severity of disease in MPP.

## Materials and methods

### Ethical approval

The study was approved by the Ethical Committee of Shenzhen Children’s Hospital under registration number 2016013. The guardians of the recruited children provided their informed consent. All procedures performed in this study were in accordance with the ethical standards of the institutional and/or national research committee, as well as with the 1964 Helsinki declaration and its subsequent amendments or comparable ethical standards.

### Sample inclusion and pathogen examination

All children with pneumonia were enrolled from inpatients at Shenzhen Children’s Hospital. The inclusion criteria for patients were as follows: 1) no asthma; 2) a diagnosed *M. pneumoniae* infection via BALF detection; 3) no antibiotic exposure for at least 1 month before sampling; 4) no admission to a pediatric intensive care unit (PICU); and 5) no mechanical ventilation during hospitalization. Age-matched healthy children were recruited after physical examination in Shenzhen Children’s Hospital according to the following criteria: 1) no asthma or family history of allergies; 2) no history of pneumonia; 3) no wheezing, fever, cough or other respiratory/allergic symptoms at sampling one month prior to the study; 4) no antibiotic exposure within 1 month; and 5) no disease symptoms one week after sampling.

URT and LRT sampling was conducted 3 to 11 days after hospitalization when the diseased children maintained a high fever or had no significant disease remission (Table S1). URT samples were collected by NP (25-800-A-50, Puritan, Guilford, North Carolina, USA) and OP (155 C, COPAN, Murrieta, California, USA) swabs, and BALF was collected by fiberoptic bronchoscopy. The same BALF sample was used for both clinical pathogen detection and 16 S rRNA gene sequencing. All samples were frozen at –80 °C within 10 minutes of sample collection.

Common pathogens were detected by the following methods: bacterial culturing was conducted to detect clinical common bacterial pathogens^25^, and the nucleic acid testing (NAT) method was applied to identify the viral or atypical pathogens as described previously^26^. Unused swabs and DNA extraction kits were utilized as negative controls to assess experimental contamination^26^.

### DNA extraction, library construction, and sequencing

The genomic DNA of the microbiota was extracted using the Power Soil DNA Isolation Kit (Mo Bio Laboratories, Carlsbad). A PCR amplicon library was constructed using the V3-V4 hypervariable region of the 16 S rRNA gene^14^. The qualified libraries were then sequenced with the Illumina MiSeq sequencing platform (Illumina, San Diego, USA)^27^.

### Data processing, statistical analysis, and visualization

Raw sequencing data were processed with the QIIME pipeline^28^ and in-house scripts^26^. Low-quality data filtration, OTU clustering, representative taxon classification and community diversity calculation were conducted as described previously^26, 29^. PERMANOVA was used to evaluate the confounding effect of subject characteristics on microbiota composition^30, 31^. An nMDS plot was used to visualize the concordance of the overall microbial community composition between niches. Inter-group comparisons were performed via the Wilcoxon rank-sum test. Hierarchical clustering of NP, OP and BALF microbial samples in MPP patients was conducted, and the sample backgrounds in different sub-clusters were summarized. Differences in the top fifteen genera in the NP, OP and BALF microbiota were calculated by Kruskal–Wallis tests, and all multiple test results were adjusted by the FDR method. The intra-individual distance between the BALF and NP/OP microbiota was calculated by the Bray-Curtis dissimilarity. All graphs were produced by the package ‘ggplot2’ of the R software (v3.2.3).

### Data availability

All the sequencing data have been deposited in GenBank under the accession numbers SRP090593 (healthy children) and SRP130820 (children with pneumonia).

## Electronic supplementary material

Figure S3

Figure S1

Figure S2

Supplementary Tables
