# Supplementary figures and images for: The concordance between upper and lower respiratory microbiota in children with Mycoplasma pneumoniae pneumonia
Source: Emerg Microbes Infect. 2018 May 23;7:92. doi: 10.1038/s41426-018-0097-y (PMC5964150; doi:10.1038/s41426-018-0097-y)

Relative Abundance(log10)

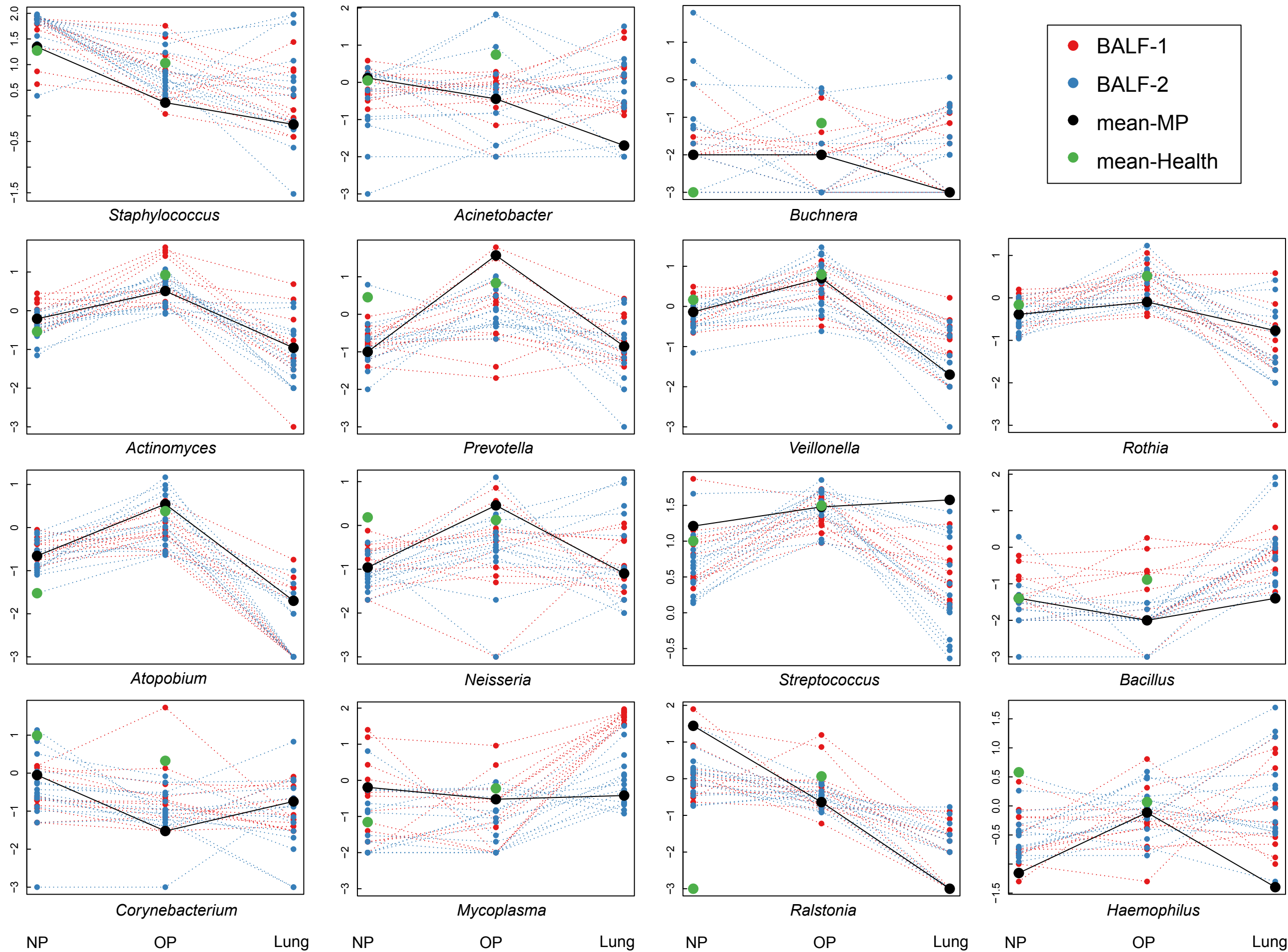

Supplement: Supplementary file 1 — Figure S3 [file 41426_2018_97_MOESM1_ESM.pdf]

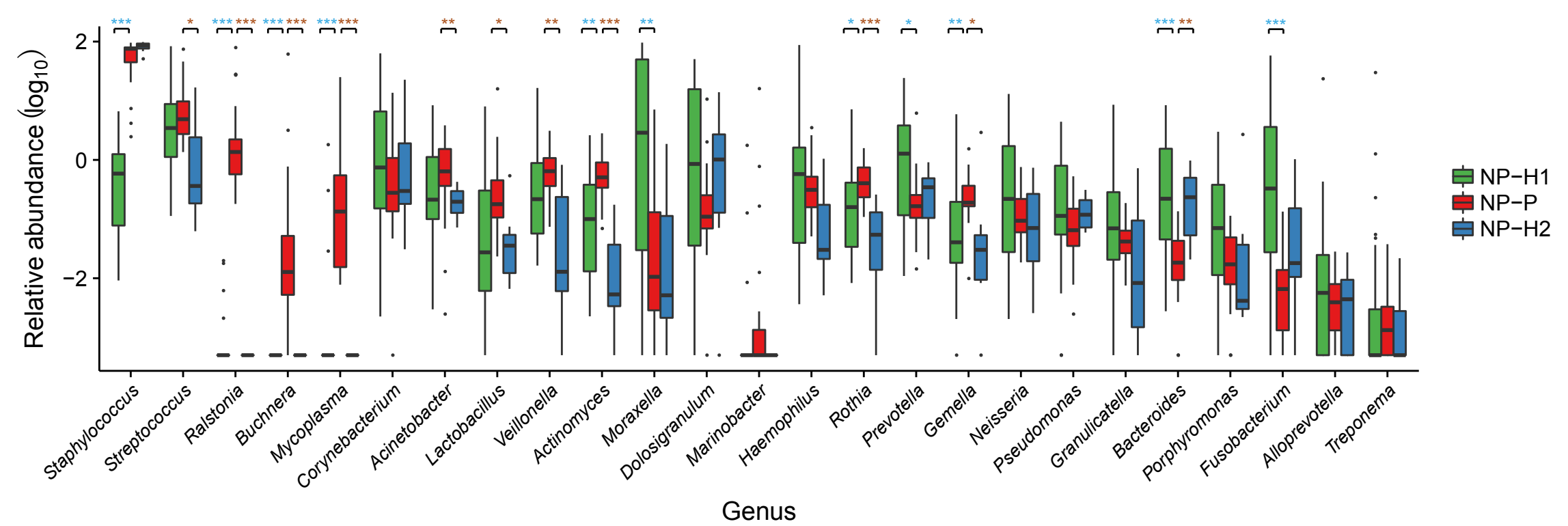

Supplement: Supplementary file 2 — Figure S1 [file 41426_2018_97_MOESM2_ESM.pdf]

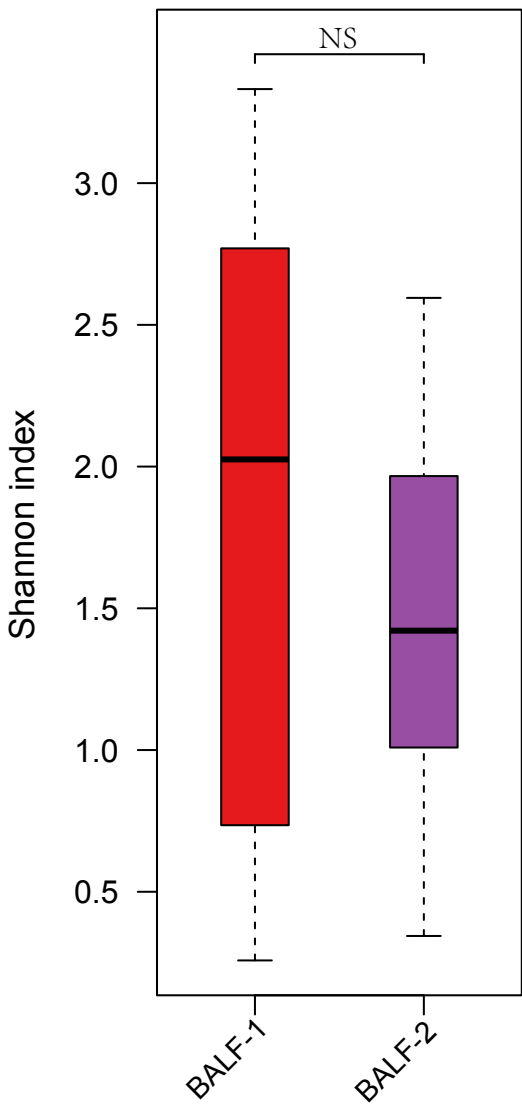

Supplement: Supplementary file 3 — Figure S2 [file 41426_2018_97_MOESM3_ESM.pdf]
